# The Conundrum of Treating Portal Vein Thrombosis and Submucosal Esophageal Hematoma

**DOI:** 10.7759/cureus.12031

**Published:** 2020-12-11

**Authors:** Davinder Singh, Kamran Zaheer, Varun Dobariya, Alexis P Lester, Samson Teka

**Affiliations:** 1 Internal Medicine, Joan C. Edwards School of Medicine - Marshall University, Huntington, USA

**Keywords:** submucosal, hematoma, esophageal, anticoagulant, heparin drip, portal vein thrombosis

## Abstract

Submucosal esophageal hematoma (SEH) is an uncommon clinical entity and a rare form of esophageal insult. Patients usually present with retrosternal chest pain and dysphagia, which often make the diagnosis of SEH difficult as it mimics common cardiovascular and pulmonary disorders. One of the common inciting factors includes the use of anticoagulants. In this report, we discuss the case of a patient with portal vein thrombosis who was treated with heparin and consequently developed SEH.

## Introduction

Hematomas of the esophagus are a rare part of a spectrum of injuries. Some of the common esophageal complications are tears (Mallory-Weiss syndrome) and esophageal ruptures (Boerhaave syndrome) [1]. Making the correct diagnosis in these patients is very important as symptoms may mimic other common differentials such as myocardial infarctions or pulmonary thromboembolism. Other differential diagnoses include spontaneous rupture of the stomach, acute pericarditis, pneumothorax, dissecting aneurysms, and pancreatitis [2]. Recognizing clinical signs early can not only help with narrowing the diagnosis but also prevents worsening complications such as bleeding and perforation. The classical triad of symptoms includes hematemesis, chest pain, and sudden dysphagia or odynophagia [3]. We discuss a patient who presented with two of the three classical symptoms. With the increasing use of multiple antiplatelet agents and the increased risk for esophageal hematoma formation, clinicians should be aware of the condition and avoid misdiagnosis. More specifically, patients with cirrhosis complicated with portal vein thrombus are placed on anticoagulation to prevent clot extension. In this report, we present a difficult case where submucosal esophageal hematoma (SEH) was a byproduct of treating portal vein thrombus with heparin.

## Case presentation

**Figure 1:**
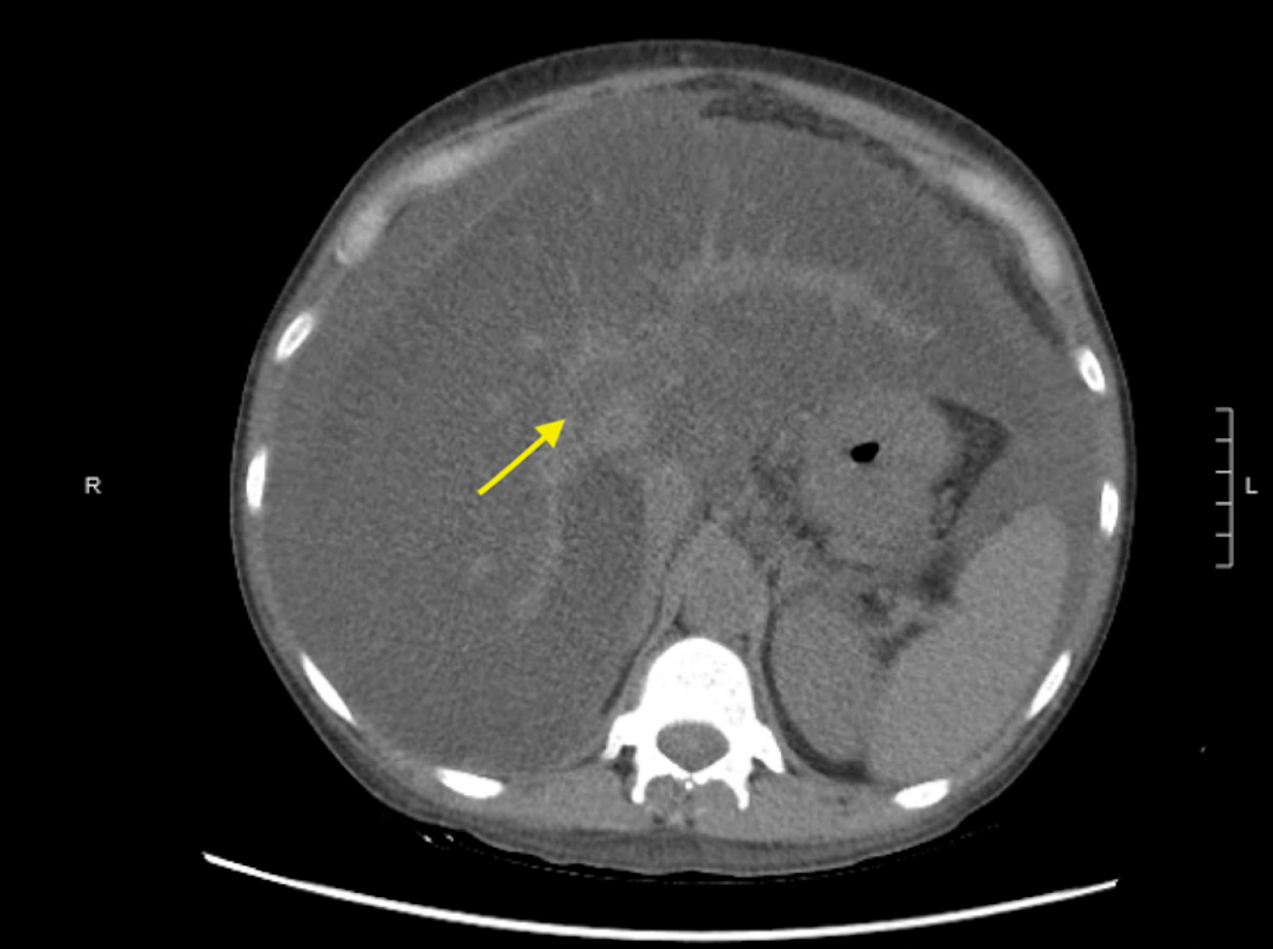
CT of the abdomen without contrast displaying portal vein thrombosis (arrow) CT: computed tomography

**Figure 2:**
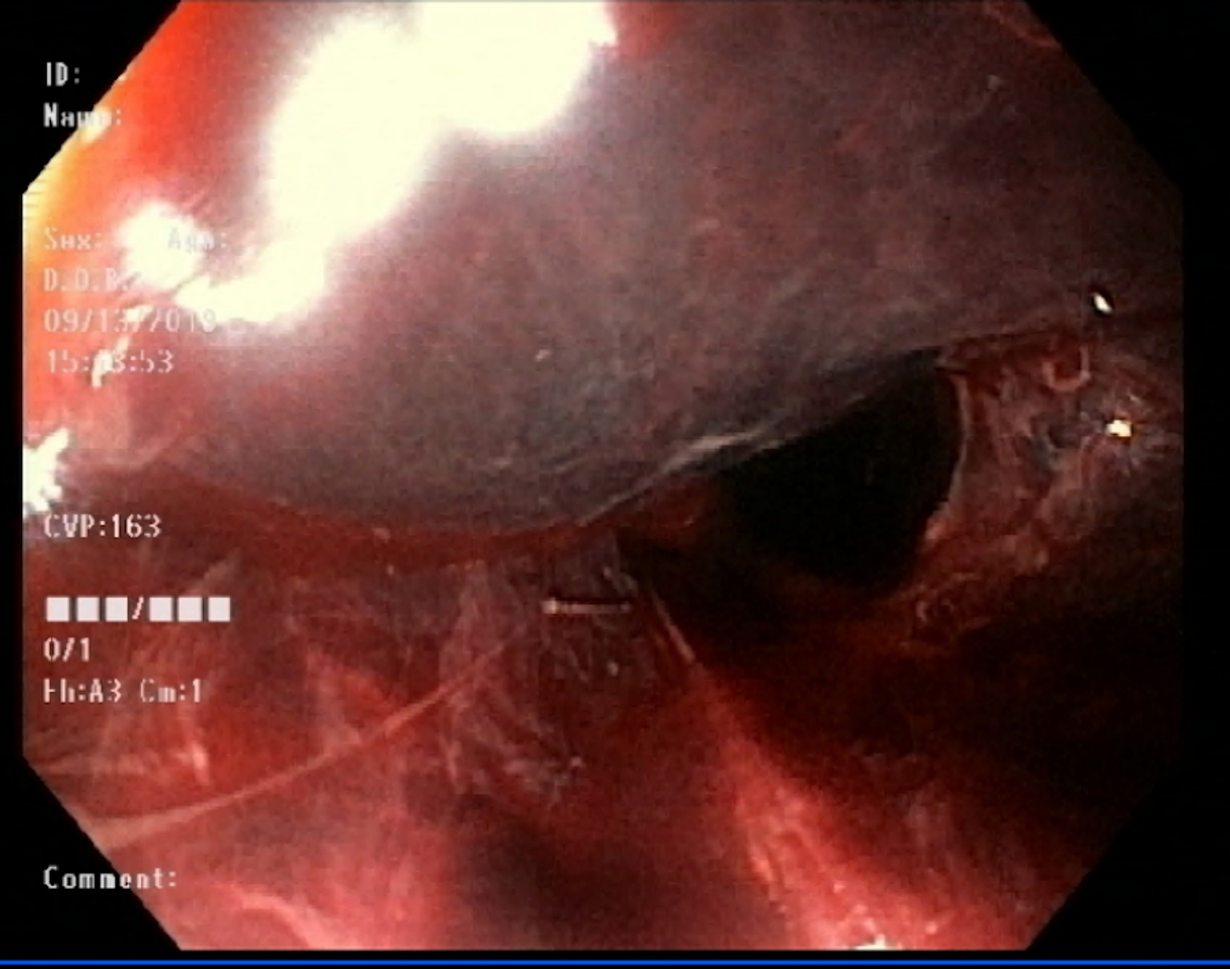
Esophagogastroduodenoscopy showing esophageal submucosal hematoma

A 42-year-old female presented to the hospital with increased abdominal swelling and bilateral leg swelling for one month. The patient’s past medical history was significant for cirrhosis secondary to alcohol abuse, intravenous drug use (IVDU), hepatitis C, hypersensitivity lung disease (HLD), and pancreatitis. Initial labs were remarkable for elevated prothrombin time, international normalized ratio (INR), total bilirubin, leukocytosis, and lactic acid. She was started on piperacillin-tazobactam (Zosyn®) for coverage of possible spontaneous bacterial peritonitis. Initial CT imaging revealed portal vein thrombosis (Figure 1) and the patient was consequently started on a heparin drip. The following day, the patient reported melena and episodes of hematemesis, and heparin was discontinued. Next, the patient was transfused with 2 units of packed red blood cells and started on octreotide and pantoprazole drip. Her hemoglobin improved from 6.6 to 7.4. Due to the ongoing bleeding, the patient underwent an endoscopy, which revealed SEH without variceal bleeding (Figure 2).

**Figure 3:**
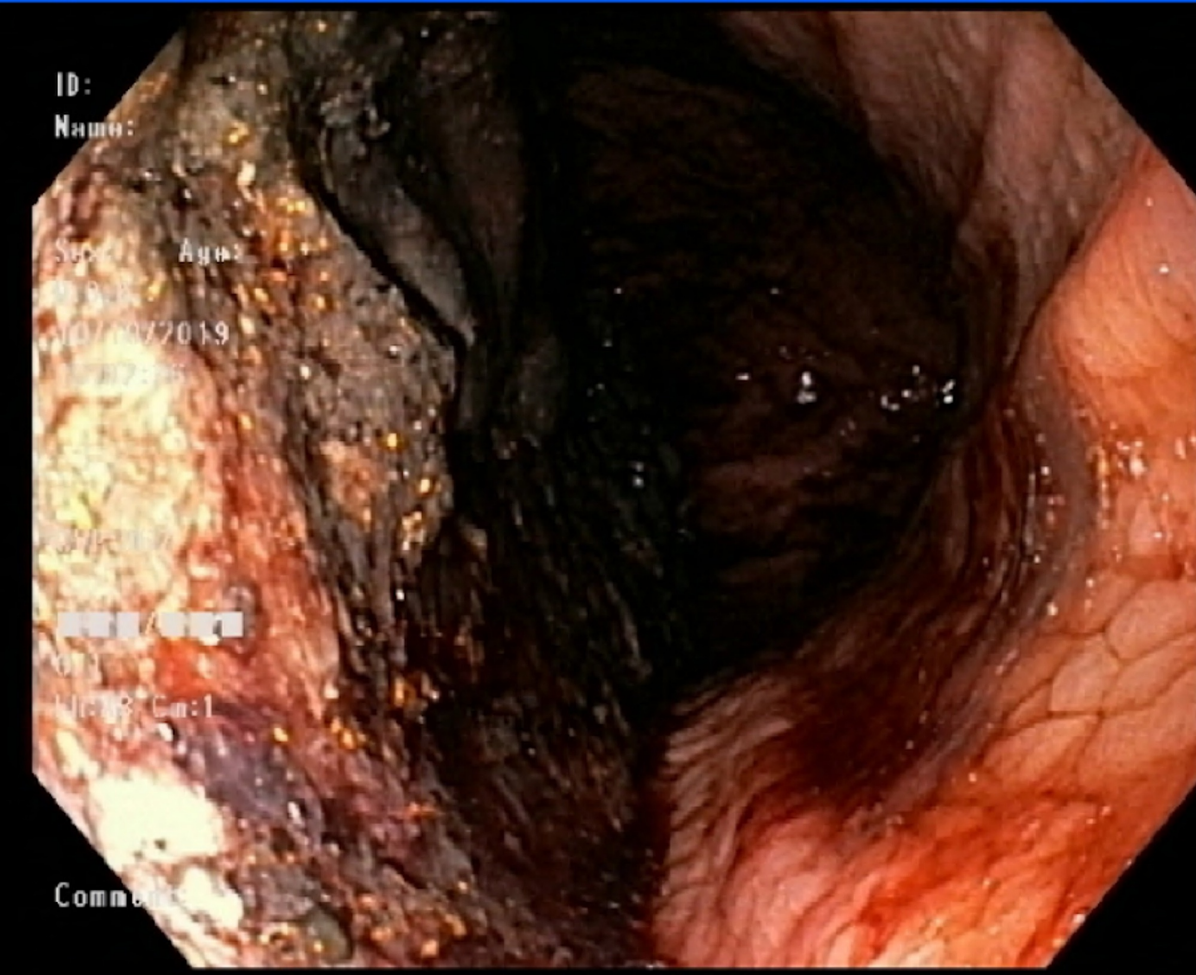
Repeat esophagogastroduodenoscopy showing no bleeding in the esophagus

**Figure 4:**
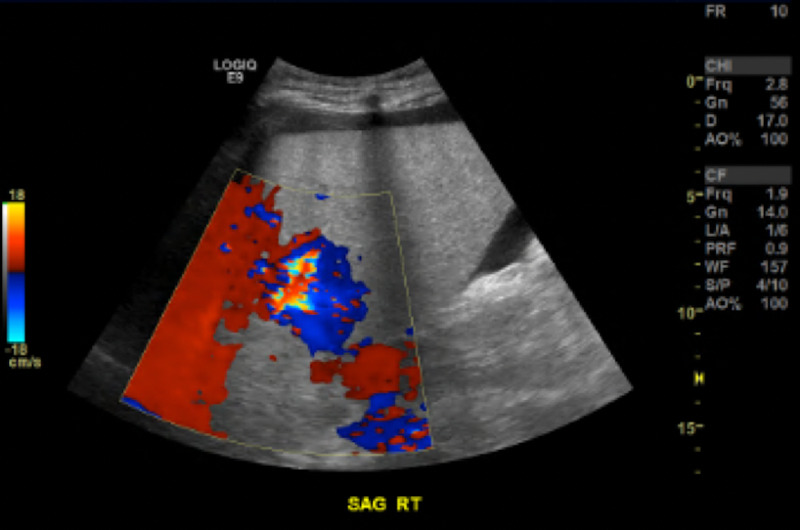
Bidirectional blood flow in the portal vein with no indication of thrombosis

The patient's hemoglobin on admission was 13.6, and on the day of episodes of hematemesis, it was 7.4. The gastroenterologist placed a nasogastric (NG) tube for nutrition via endoscopy. Following the above measures, the patient became hemodynamically stable and the bleeding was stopped. Repeat abdominal CT after conservative measures showed no signs of bleeding in the esophagus (Figure 3). Repeat abdominal ultrasound showed no indications of thrombosis and displayed a patent portal vein with bidirectional blood flow (Figure 4).

## Discussion

A hematoma of the esophagus is a rare condition characterized by a collection of blood in the wall of the esophagus. Clinical signs of SEH are unreliable and hence require further investigation. CT scans can reveal the intramural soft tissue density seen in hematomas [4]. Other imaging modalities such as esophageal swallowing study or upper gastrointestinal (GI) endoscopy are used for further confirmation of SEH or for inconclusive CT results. SEH can be further classified into four stages where stage I represents an isolated hematoma and stage II is characterized by hematoma being surrounded by tissue edema. In stage III, the hematoma with edema physically compresses the esophageal lumen, which causes respiratory difficulty leading to intubation. Stage IV is defined by the hematoma obliterating the esophageal lumen [4]. Our patient had stage III SEH, and due to early detection, she was managed conservatively and discharged home safely.

Classical clinical symptoms of SEH consist of retrosternal pain, dysphagia, and upper GI bleeding. It is believed that excessive anticoagulation and mechanical forces of retching account for the majority of the cases [5]. Our patient was on a heparin drip and had hematemesis early during her hospital course. The use of anticoagulants or antiplatelet therapy can cause hematomas in all parts of the GI tract including the esophagus, stomach, duodenum, and cecum [4]. Even though hematomas are considered benign, correct diagnosis is important since the symptoms mimic other common chest pain pathologies. Endoscopic evaluation can provide a reliable method for diagnosing hematomas since they have a distinctive endoscopic appearance. Submucosal hematomas involve the distal esophagus and often extend into the middle third and occasionally the upper third of the esophagus [6,7]. Another differentiating factor is the inclusion of the female sex. Esophageal hematomas predominantly affect females (80%) while Mallory-Weiss syndrome (80%) and Boerhaave syndrome (60-78%) mainly affect males [8,9]. While Mallory-Weiss syndrome can also present with small submucosal hematomas, they often have a different location and characterization. The submucosal hematomas seen in Mallory-Weiss are localized and do not spread beyond 1-2 cm, which is explained by the mucosal tear decompressing the hematoma [10].

Misdiagnosis can result in excessive use of anticoagulant therapy resulting in worsening of bleeding. Our patient initially presented with abdominal swelling and pain, and after a proper diagnostic workup, she was placed on heparin for portal vein thrombosis. The anticoagulant may have caused active bleeding leading to hematemesis and melaena and further complicated her condition. We determined that the patient had SEH only after she was endoscopically examined. The patient was kept on conservative treatment until her vitals stabilized and repeat imaging confirmed the resolution of SEH and patent portal vein.

## Conclusions

While diagnosing esophageal hematomas can prove to be difficult, early recognition and detection can help improve hospital stay and prevent further complications. Cardiac and pulmonary causes of the chest and abdomen present difficulty in distinguishing GI symptoms. Various imaging modalities provide invaluable information on the location, size, and extent of the hematomas. An accurate diagnosis of the hematoma can help physicians avoid giving anticoagulants and performing unnecessary tests that can lead to perforation or bleeding. By providing patients with conservative treatment early in their hospital stay, surgery can be prevented.
